# Early onset muscle weakness and disruption of muscle proteins in mouse models of spinal muscular atrophy

**DOI:** 10.1186/2044-5040-3-24

**Published:** 2013-10-11

**Authors:** Justin G Boyer, Lyndsay M Murray, Kyle Scott, Yves De Repentigny, Jean-Marc Renaud, Rashmi Kothary

**Affiliations:** 1 Ottawa Hospital Research Institute, Regenerative Medicine Program, 501 Smyth Road, Ottawa, ON K1H 8L6, Canada; 2 Department of Cellular and Molecular Medicine, University of Ottawa, Ottawa, ON K1H 8M5, Canada; 3 Department of Medicine, University of Ottawa, Ottawa, ON K1H 8M5, Canada

**Keywords:** Motor neuron disease, Skeletal muscle, Sodium channels, Ryanodine receptors, SERCA, Spinal muscular atrophy, Survival motor neuron

## Abstract

**Background:**

The childhood neuromuscular disease spinal muscular atrophy (SMA) is caused by mutations or deletions of the survival motor neuron (*SMN1*) gene. Although SMA has traditionally been considered a motor neuron disease, the muscle-specific requirement for SMN has never been fully defined. Therefore, the purpose of this study was to investigate muscle defects in mouse models of SMA.

**Methods:**

We have taken advantage of two different mouse models of SMA, the severe *Smn*^*-/-*^;*SMN2* mice and the less severe *Smn*^*2B/-*^ mice. We have measured the maximal force produced from control muscles and those of SMA model mice by direct stimulation using an *ex vivo* apparatus. Immunofluorescence and immunoblot experiments were performed to uncover muscle defects in mouse models of SMA. Means from control and SMA model mice samples were compared using an analysis of variance test and Student’s *t* tests.

**Results:**

We report that tibialis anterior (TA) muscles of phenotype stage *Smn*^*-/-*^;*SMN2* mice generate 39% less maximal force than muscles from control mice, independently of aberrant motor neuron signal transmission. In addition, during muscle fatigue, the *Smn*^*-/-*^;*SMN2* muscle shows early onset and increased unstimulated force compared with controls. Moreover, we demonstrate a significant decrease in force production in muscles from pre-symptomatic *Smn*^*-/-*^;*SMN2* and *Smn*^*2B/-*^ mice, indicating that muscle weakness is an early event occurring prior to any overt motor neuron loss and muscle denervation. Muscle weakness in mouse models of SMA was associated with a delay in the transition from neonatal to adult isoforms of proteins important for proper muscle contractions, such as ryanodine receptors and sodium channels. Immunoblot analyses of extracts from hindlimb skeletal muscle revealed aberrant levels of the sarcoplasmic reticulum Ca^2+^ ATPase.

**Conclusions:**

The findings from this study reveal a delay in the appearance of mature isoforms of proteins important for muscle contractions, as well as muscle weakness early in the disease etiology, thus highlighting the contributions of skeletal muscle defects to the SMA phenotype.

## Background

With an overall carrier frequency of 1:40, spinal muscular atrophy (SMA) is a major leading genetic cause of infant deaths, affecting 1 in 6,000 to 10,000 births [1-3]. Spinal muscular atrophy is an autosomal recessive disorder traditionally classified into different types based on the clinical severity of the symptoms [4]. In 1995, the SMA-determining gene was identified and named '*survival motor neuron*’ (*SMN*) [5]. This gene is located on chromosome 5q13 in humans, in a region containing an inverted duplication of 500 kilobase pairs. This results in two virtually identical copies of the *SMN* gene; *SMN1* and *SMN2*[5-8].

In the mouse, the *Smn* gene is present as a single copy, and homozygous loss of function leads to a pre-implantation lethality [9]. However, when the *Smn* knockout is coupled with low levels of human SMN expressed from a *SMN2* transgene, a severe phenotype approximating type I SMA is observed in *Smn*^*-/-*^;*SMN2* mice [10]. Since this original discovery, several other mouse models of SMA have been generated, including a milder model termed *Smn*^*2B/-*^. These latter mice do not harbor the *SMN2* transgene but rather harbor one null allele and a second allele with a 3-nucleotide substitution in the exonic splice enhancer of exon 7 of the mouse *Smn* gene (*2B* mutation) [11]. *Smn*^*2B/-*^ model mice display a milder SMA phenotype, owing to slightly higher Smn protein levels than the severe model [12].

Motor neuron cell loss and muscle denervation are considered two pathological hallmarks of SMA. Exactly how SMN depletion leads to motor neuron degeneration is unclear and remains the focus of intense research. In addition, recent advances in the field have highlighted the involvement of other tissues in the pathophysiology of SMA, of which skeletal muscle appears to be an important candidate [4,13].

In *Drosophila*, Smn was discovered to be a sarcomeric protein interacting with α-actinin, a cross-linking protein that stabilizes actin microfilaments [14]. Walker and colleagues later confirmed these findings and specifically identified Smn as a Z-disc component in skeletal and cardiac muscle of mice [15]. At present, the function of Smn at this adhesion site is unknown but Smn is likely to have a specific function other than snRNP biogenesis in muscle [15]. An Smn interacting protein screen in C2C12 myoblasts suggests that the function of Smn in muscle is dynamic and probably differs during varying stages of myogenesis based on its protein interactome [16]. A proteomic screen performed by Mutsaers *et al.* identified an increase in proteins involved in programmed cell death in pre-symptomatic *Smn*^*-/-*^;*SMN2* mice [17]. Several reports have highlighted the possibility of delayed myogenesis in mouse models of SMA. The basis of this notion comes from muscle morphological studies demonstrating a lack of increase in myofiber size and the increased levels of embryonic and neonatal myosin heavy chain (MHC) isoforms [18-20]. However, it is not known whether or how impaired muscle growth contributes to muscle weakness in SMA, since at present no comprehensive analysis has been performed relating to muscle force production in mouse models of SMA.

Here, we show previously unreported pathophysiological muscle defects in severe (*Smn*^*-/-*^;*SMN2*) and less severe (*Smn*^*2B/-*^) mouse models of SMA. We report pronounced muscle weakness in these mice. These observations were associated with altered expression of proteins that are developmentally regulated and are important for proper physiological muscle function. Furthermore, we show that muscle weakness is an early feature, observed prior to any overt motor neuron loss and muscle denervation in mouse models of SMA. Thus, we conclude that muscle defects contribute to the phenotype in SMA mouse models. Uncovering skeletal muscle defects in the context of SMA is of the utmost importance to better understand the SMA phenotype and for the development of targeted therapeutics.

## Methods

### Mouse models

The *Smn*^*-/-*^;*SMN2* (Jackson Labs, Bar Harbor, ME, USA) and *Smn*^*2B/-*^[12] mice were housed and cared for according to the Canadian Council on Animal Care guidelines and the University of Ottawa Animal Care Committee protocols. Tissues from pre-symptomatic mice were collected at postnatal day (P) 2 for severe *Smn*^*-/-*^;*SMN2* mice, and P9 for *Smn*^*2B/-*^ mice. Tissues were also collected from phenotype stages at P5 for *Smn*^*-/-*^;*SMN2* and P21 for *Smn*^*2B/-*^ mice. Muscles used for RNA and protein analysis were flash frozen in liquid nitrogen and stored at -80°C.

### Hindlimb denervation

Denervation surgeries were performed in accordance with the guidelines set by the Canadian Council on Animal Care. Young mice (P14) were anaesthetized by inhalation of isoflurane. Experimental denervation was achieved by revealing the sciatic nerve and removing 2 to 3 mm of the nerve in the thigh section to cease neural input and prevent nerve regrowth. A sham procedure was performed in parallel to serve as control; this consisted of exposing the mice to identical experimental conditions except for cutting the nerve. The TA muscles were collected and flash frozen from denervated and sham operated mice one and seven days following surgery.

### Immunoblotting

Total tissue lysate extract was obtained by grinding flash frozen tissues in a liquid nitrogen pre-cooled mortar and pestle. The concentration of each sample was determined by Bradford assay. Samples were subjected to sodium dodecyl sulfate polyacrylamide gel electrophoresis and examined by immunoblot, as previously described [16]. Primary antibodies used were: calsequestrin (Abcam, Toronto, ON, Canada), glyceraldehyde-3-phosphate dehydrogenase (GAPDH, Abcam, Toronto, ON, Canada), Na_v_1.4 (Alomone, Jerusalem, Israel), Na_v_1.5 (Alomone, Jerusalem, Israel), nuclear factor 1 (Abcam, Toronto, ON, Canada), sarcoplasmic reticulum Ca^2+^ ATPase (SERCA1a, Cell Signaling, Danvers, MA, USA), and zinc-finger E box-binding protein (ZEB) (Novus Biologicals, Littleton, CO, USA). Signals were detected using enhanced chemiluminescence (Thermo, Florence, KY, USA). Densitometric analyses were performed using ImageJ software (NIH). Immunoblot data were normalized to GAPDH levels to control for possible loading differences.

### Force measurements and fatigue protocol

The TA muscles were dissected from P2 and P5 control and severe *Smn*^*-/-*^;*SMN2* mice, and from P9 control and *Smn*^*2B/-*^ mice. Muscles were constantly immersed in physiological saline solution containing 118.5 mM NaCl, 4.7 mM KCl, 2.4 mM CaCl_2_, 3.1 mM MgCl_2_, 25 mM NaHCO_3_, 2 mM NaH_2_PO_4_, and 5.5 mM D-glucose. Solutions were continuously bubbled with 95% O_2_, 5% CO_2_ for a pH of 7.4. Solutions containing 30 μM of tubocurarine hydrochloride pentahydrate (Sigma, Oakville, ON, Canada) were prepared by adding the appropriate amount directly to the physiological solution. The flow of physiological solution below and above muscles was maintained at a total of 15 ml/min and a temperature of 37°C. Tetanic contractions were elicited with electrical stimulations applied across two platinum wires (4 mm apart) located on opposite sides of the muscle. Electrodes were connected to a Grass S88 stimulator and a Grass SIU5 isolation unit (Grass Technologies/Astro-Med Inc., Warwick, RI, USA). Tetanic contractions were elicited with 200 ms trains of 0.3 ms, 12 V (supramaximal voltage) pulses at a frequency of 200 Hz. For all experiments, muscle length was adjusted to achieve maximal force production and muscles were allowed a 30 min equilibration period during which a tetanic contraction was elicited every second. Maximal force production was determined by increasing frequencies from 1 to 200 Hz. Muscles were then fatigued by increasing the contraction rate to one contraction per second for 180 s. Twitch (obtained when stimulated with one square pulse) or tetanic force was defined as the force that developed during stimulation and was calculated as the difference between the maximum force during contraction and the force measured 5 ms before the contraction. Unstimulated force was defined as the force generated by muscles in the absence of electrical stimulation and was observed during fatigue when muscles failed to relax between contractions; it was calculated as the difference in the baseline 5 ms before a contraction and the baseline 5 ms before fatigue was elicited. Muscle weight and length were used to calculate the cross-sectional area of the muscle that was used to normalize force measurements in each experiment.

### RNA isolation

Total RNA was isolated from skeletal muscle tissue using a homogenizer and the RNeasy kit (Qiagen, Toronto, ON, Canada) according to the manufacturer’s instructions. RNA samples were treated with DNase (gDNA wipeout buffer, Qiagen, Toronto, ON, Canada) to eliminate DNA contamination and concentrations were determined using a Nanophotometer spectrophotometer (MBI Lab Equipment, Dorval, QC, Canada).

### Reverse-transcription polymerase chain reaction (RT-PCR)

RNA was reverse-transcribed using the quantitect reverse-transcription kit (Qiagen, Toronto, ON, Canada). Primer sequences and PCR conditions used to detect the spliced variants of the ryanodine receptor *RyR1* gene were identical to those previously described [21]. A negative control in which water was added instead of cDNA was prepared in parallel for every PCR. Quantification of the RT-PCR results was achieved using ImageJ software.

### Immunofluorescence

Neuromuscular junction (NMJ) immunofluorescence and quantification was performed as described previously [22]. Post-synaptic acetylcholine receptors were labeled with α-bungarotoxin (Molecular Probes, Burlington, ON, Canada) while the pre-synaptic terminal was labeled with anti-neurofilament and anti-synaptic vesicle protein 2 (both from Developmental Studies Hybridoma Bank, Iowa City, IA, USA). All secondary antibodies were purchased from Jackson Labs. Immunofluorescence images were captured using a Zeiss Confocal microscope (LSM 510 Meta DuoScan, Toronto, ON, Canada). For each muscle, four to six fields of view were quantified and a total of counted endplates ranging between 99 and 263 were included per animal in the analysis.

### Histological analysis

The lumbar (L1 and L2) region of the spinal cord was collected from control, pre-symptomatic *Smn*^*-/-*^;*SMN2* and *Smn*^*2B/-*^ mice. Tissues were fixed in 4% paraformaldehyde for 24 hrs, embedded in paraffin, cut into sections (10 μm) and stained with hematoxylin and eosin (H & E). Motor neurons were identified by their shape and size within the ventral horn region of the spinal cord. Motor neuron quantification was performed on every fifth section within the L1 and L2 region on three mice from each genotype. Histological analyses were also performed on cross-sections (10 μm) from frozen TA muscles of P2 *Smn*^*-/-*^;*SMN2*, and P9 *Smn*^*2B/-*^ mice. Sections were stained with H & E using a standard protocol, images were taken with a Zeiss Axioplan2 microscope, and the myofiber area was calculated using ImageJ software. Approximately one thousand fibers were counted for each genotype analyzed.

### Statistical analyses

Data are presented as the mean ± standard error of the mean. Analysis of variance (Statistical Analysis Software Institute Inc., Cary, NC, USA) was used to determine significance in the fatigue data. A Student’s *t* test was performed using MS Excel to compare the means of all other data. Significance was set at *P* < 0.05.

## Results

### Skeletal muscle weakness in *Smn*^-/-^;*SMN2* mice

**Figure 1:**
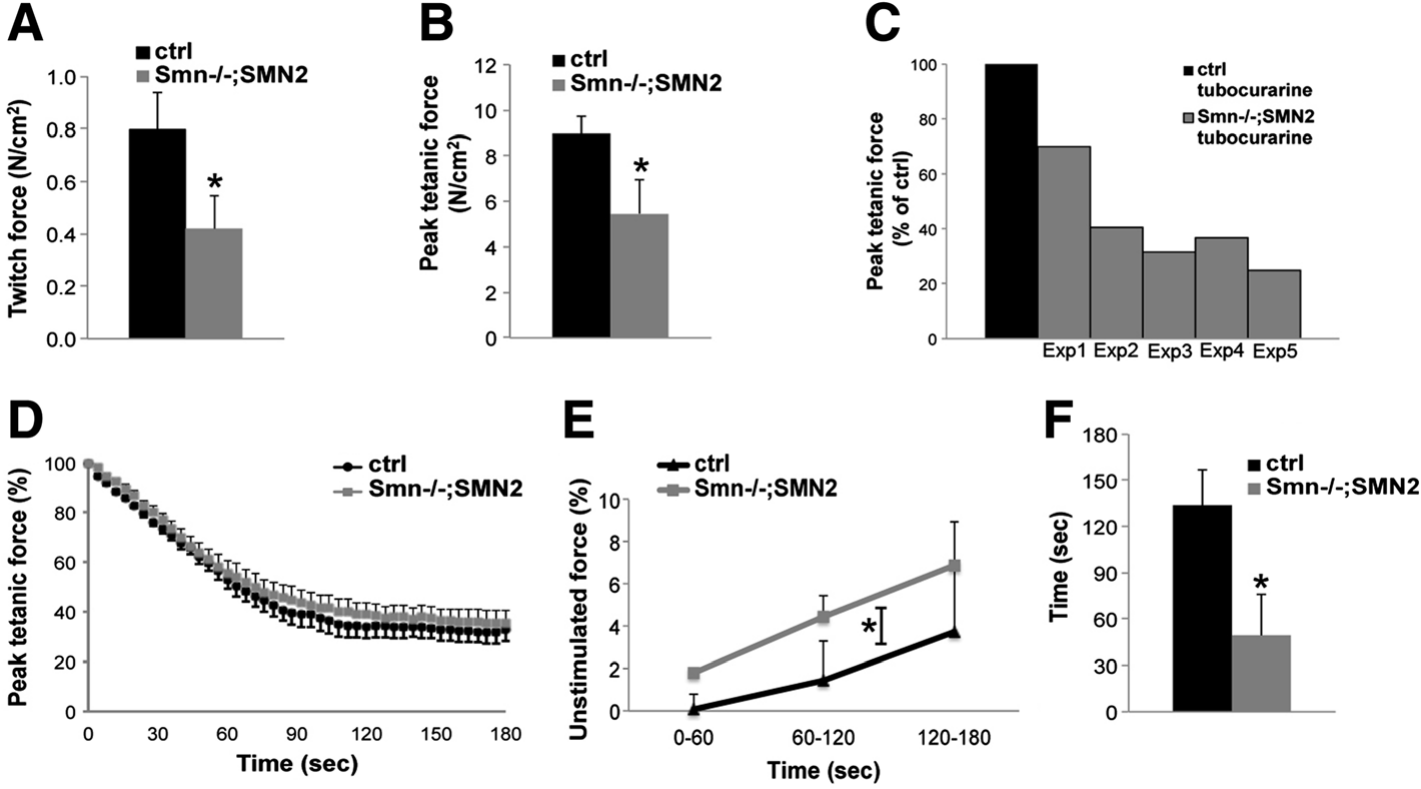
**Muscle weakness in muscle from *Smn***^***-/-***^**;*SMN2 *mice. (A)** TA muscle preparations from P5 *Smn*^*-/-*^;*SMN2* mice and control littermates were used to assess tetanic and twitch force normalized to the muscle cross-sectional area. P5 *Smn*^*-/-*^;*SMN2* TA muscles produce significantly less twitch force than control littermates. **(B)** Reduction in normalized maximal peak tetanic force in P5 *Smn*^*-/-*^;*SMN2* TA muscle compared with controls. **(C)** Administration of tubocurarine to block NMJs did not influence relative force production in *Smn*^*-/-*^;*SMN2* muscles. In five independent experiments, *Smn*^*-/-*^;*SMN2* mice produced less force than controls following treatment with tubocurarine. **(D)** Similar relative force decreases in *Smn*^*-/-*^;*SMN2* and control mice during fatigue elicited with one tetanic contraction every second for 3 min. Peak tetanic forces are expressed as a percentage of the pre-fatigue force. **(E)** Unstimulated force occurred when muscles failed to relax between contractions and is expressed as a percentage of the pre-fatigue peak tetanic force. During a fatigue protocol, P5 *Smn*^*-/-*^;*SMN2* TA muscles show increased unstimulated force compared with controls. **(F)** Unstimulated force appeared much sooner in *Smn*^*-/-*^;*SMN2* TA muscles than in controls. NMJ, neuromuscular junction; TA, tibialis anterior; *N* = 5 or 6; *, *P* < 0.05.

To date, no physiological study has been performed on muscles from severe SMA model mice. To this end, we have analyzed the twitch and peak tetanic force produced by direct stimulation of TA muscles of *Smn*^*-/-*^;*SMN2* mice and control littermates at P5. To account for variations in muscle size, all force values were normalized to muscle cross-sectional area. Compared with control muscles, *Smn*^*-/-*^;*SMN2* mice produced 47% less twitch force, as measured after one stimulation, and 39% less maximum peak tetanic force, as measured at 200 Hz (Figure 1A,B).

Aberrant NMJ morphology and function have previously been highlighted in several mouse models of SMA [20,22-24]. In isolated muscle preparations, many fibers receive an indirect stimulation via the remaining nerve stump. Although it has previously been demonstrated that the TA muscle is fully innervated in SMA model mice [20,25], one possible explanation for the observed decrease in force is that the applied stimulus enters the residual nerve before reaching the myofibers. Thus, if poorly functioning NMJs were present in the preparation, it would negatively impact the force because fewer fibers would be stimulated. To address this possibility, we measured the maximal force production in muscles from control and mutant mice in the presence or absence of tubocurarine, which blocks acetylcholine receptors. Should aberrant NMJs negatively affect TA muscle force production, it would be expected that the relative force produced by muscles from *Smn*^*-/-*^;*SMN2* mice would be equal to the control values in the presence of tubocurarine. The force production of control muscles was not affected by the presence of tubocurarine nor did we observe an increase in force production in *Smn*^*-/-*^;*SMN2* muscles after the addition of tubocurarine to the preparation compared with non-treated muscles (data not shown). Furthermore, in five independent experiments, the force production from *Smn*^*-/-*^;*SMN2* muscle was lower than that of controls (Figure 1C). These data show that at the phenotype stage, aberrant NMJs did not impact *Smn*^*-/-*^;*SMN2* TA *ex vivo* muscle force production and that mechanisms of stimulus propagation might be compromised in muscles from *Smn*^*-/-*^;*SMN2* mice.

### *Smn*^-/-^;*SMN2* muscles respond abnormally to induced muscle fatigue

To determine whether *Smn*^*-/-*^;*SMN2* muscles respond differently to muscle fatigue, we measured the decline in force with repeated tetanic stimulation for 180 s. The decrease in peak tetanic force recorded in *Smn*^*-/-*^;*SMN2* muscles was similar to control littermates (Figure 1D). During the fatigue protocol, we also measured the unstimulated force, which is defined as the force measured 100 ms before a contraction is elicited. The TA muscles of both control and *Smn*^*-/-*^;*SMN2* P5 mice generated an increase in unstimulated force as they failed to completely relax between contractions (Figure 1E). However, the *Smn*^*-/-*^;*SMN2* muscle produced significantly more unstimulated force than control counterparts. The unstimulated force of control TA muscles had a mean time of appearance at 133 s and was equivalent to 4.4% of the pre-fatigue tetanic force by the end of the protocol (Figure 1E,F). However, for the P5 *Smn*^*-/-*^;*SMN2* TA muscles*,* the average time of appearance started significantly sooner, i.e. at 49 s, with a final mean of 8.2% (Figure 1E,F). Therefore, our results suggest the presence of a defect in *Smn*^*-/-*^;*SMN2* muscles, resulting in an inability to recover from muscle fatigue over time.

### Pre-symptomatic muscle weakness in *Smn*^-/-^;*SMN2* and *Smn*^*2B/-*^ mice

**Figure 2:**
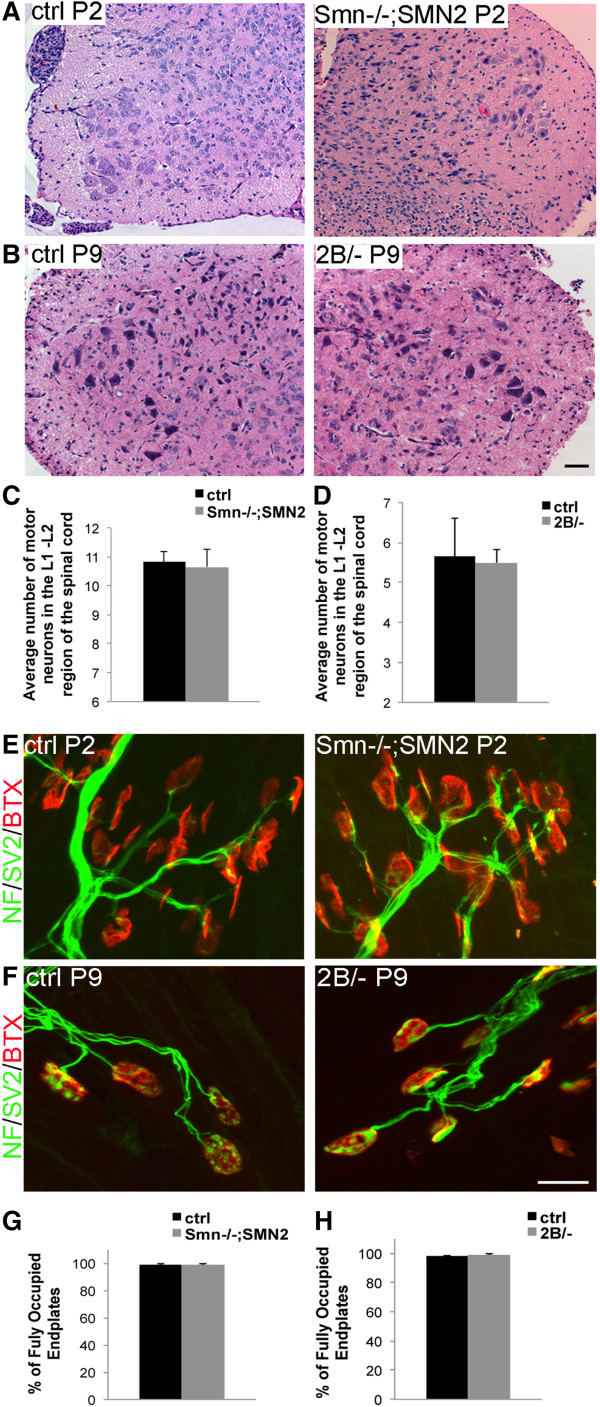
**Normal motor neuron counts and NMJ integrity in P2 *Smn***^***-/-***^**;*SMN2 *and P9 *Smn***^***2B/-***^**mice. (A, B)** Representative images of H & E staining of motor neurons in the ventral horn region of the L1 and L2 spinal cord region of P2 control and *Smn*^*-/-*^;*SMN2* mice, and P9 control and *Smn*^*2B/-*^ mice. Scale bar = 50 μm. **(C, D)** Quantification of motor neuron cell body number within the ventral horn of the lumbar (L1 and L2) region of the spinal cord for control and pre-symptomatic *Smn*^*-/-*^;*SMN2* and *Smn*^*2B/-*^ mice. **(E, F)** Representative images showing fully intact NMJs from TA muscles of control and pre-symptomatic *Smn*^*-/-*^;*SMN2***(E)** and *Smn*^*2B/-*^**(F)** mice. Post-synaptic acetylcholine receptors were labeled with α-bungarotoxin (red) while the pre-synaptic terminal was labeled with anti-NF (green) and anti-SV2 (green). Scale bar = 20 μm. **(G, H)** Quantification of the percentage of fully occupied endplates revealed no difference between control and pre-symptomatic *Smn*^*-/-*^;*SMN2* and *Smn*^*2B/-*^ mice. NF, neurofilament; NMJ, neuromuscular junction; SV2, synaptic vesicle protein 2; *N* = 3 for all experiments.

Whilst we observed a significant decrease in force production in muscles from phenotypic *Smn*^*-/-*^;*SMN2* mice, which was independent of aberrant nerve transmission in the *ex vivo* preparations, it remains possible that the muscle weakness observed could be attributed to motor neuron degeneration occurring prior to the stage of our analyses. We therefore assessed the peak tetanic force in pre-symptomatic mice. For this, we have extended our analysis to include both *Smn*^*-/-*^;*SMN2* and *Smn*^*2B/-*^ mouse models. This analysis was performed at pre-phenotypic time point of P2 and P9 in *Smn*^*-/-*^;*SMN2* and *Smn*^*2B/-*^ model mice, respectively. To confirm that these time points preceded neurodegenerative events, we assessed motor neuron number and NMJ integrity. At P2 in *Smn*^*-/-*^;*SMN2* mice, and P9 in *Smn*^*2B/-*^ mice, there was no difference in the number of motor neuron cell bodies compared with controls (Figure 2A,D). Furthermore, the percentage of fully occupied endplates was unchanged between each mouse model of SMA and the respective controls (Figure 2E,H).

**Figure 3:**
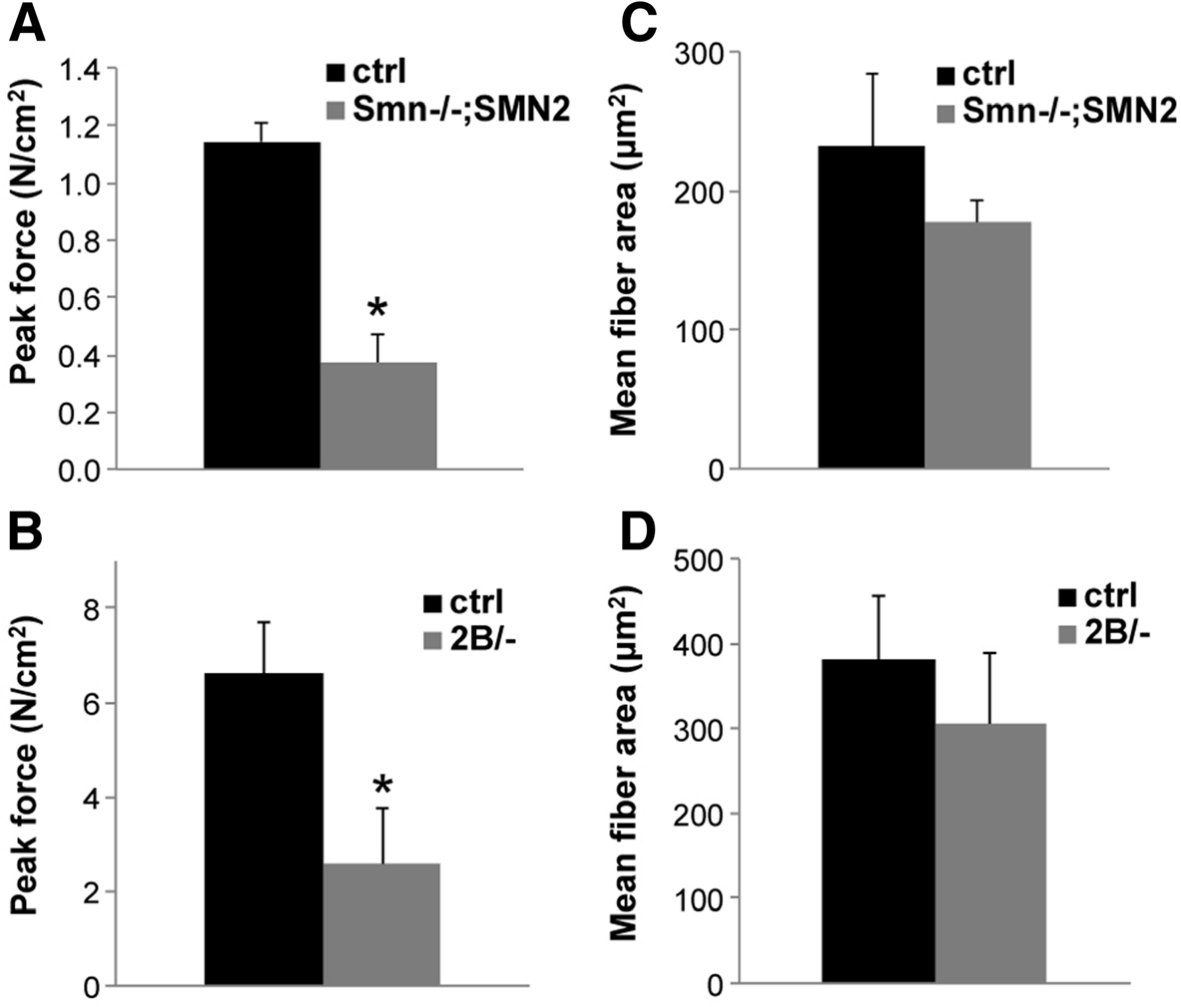
**Pre-symptomatic muscle weakness in *Smn***^***-/-***^**;*SMN2 *and *Smn***^***2B/-***^**mice. (A)** P2 *Smn*^*-/-*^;*SMN2* force measurements revealed a 67% decrease in maximal tetanic force production compared with controls. Force data were normalized to the muscle cross-sectional area. **(B)** The average peak tetanic force was reduced by 61% in P9 pre-symptomatic *Smn*^*2B/-*^ TA muscles compared with control littermates. **(C)** Mean fiber area of P2 TA muscles from *Smn*^*-/-*^;*SMN2* and control mice. **(D)** Average fiber cross-sectional area for P9 *Smn*^*2B/-*^ and control TA muscle. *N* = 3 for all experiments. *, *P* < 0.05.

Although we observed no overt motor neuron loss or denervation in both models at pre-symptomatic stage, we observed a significant decrease in peak tetanic force. TA muscles from P2 *Smn*^*-/-*^;*SMN2* mice produced 67% lower peak tetanic force than control littermates (Figure 3A). TA muscles from P9 *Smn*^*2B/-*^ mice produced 61% lower peak tetanic force than control littermates (Figure 3C). The peak forces were normalized to the cross-sectional area of each muscle; however, at this stage, we did not observe any significant difference in mean fiber area between mutant and control muscle in either *Smn*^*2B/-*^ or *Smn*^*-/-*^;*SMN2* mice (Figure 3B,D). Taken together, these data demonstrate that in two different mouse models of SMA, muscle weakness is an early feature, occurring prior to any overt motor neuron loss and denervation.

### Decreased expression of mature ryanodine receptor 1 transcripts in muscle from SMA model mice

**Figure 4:**
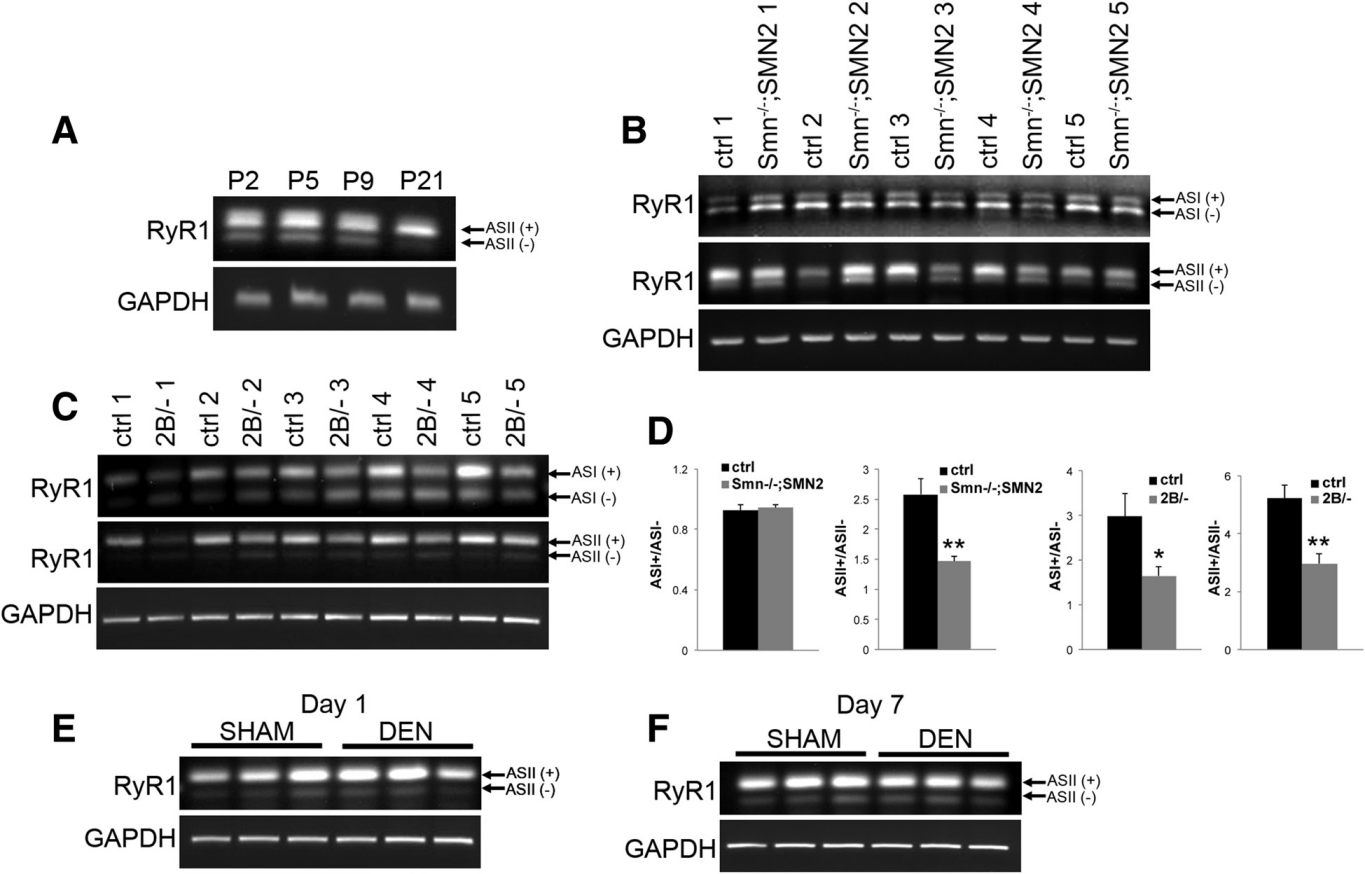
**Delayed expression of adult RyR1 mRNA splice variant in muscles from mouse models of SMA. (A)** RT-PCR on RNA from hindlimb muscle from wild type mice with primers directed against ASII (+) and ASII (-). GAPDH served as a loading control to confirm equivalence of starting cDNA levels . Note that relative ratio of ASII (+) to ASII (-) increases from P2 to P21. **(B)** RT-PCR results demonstrated no change in the expression of ASI (+) and ASI (-) variants in control and *Smn*^*-/-*^;*SMN2* samples at P5 (upper panel). However, there was decreased expression of ASII (+) and sustained expression of ASII (-) in muscle samples from P5 *Smn*^*-/-*^;*SMN2* compared with controls (middle panel). GAPDH served as a loading control. *N* = 5 for each genotype. **(C)** In control P21 mice, we observed increased expression of ASI (+) transcripts relative to ASI (-) transcripts. However in *Smn*^*2B/-*^ mice, the relative ratio of ASI (+) to ASI (-) transcripts was decreased (upper panel). Furthermore, for the ASII variant, we observed the presence of a single transcript [ASII (+)] in P21 control samples, while in *Smn*^*2B/-*^ samples, we observed a decrease in ASII (+) transcripts compared with controls. The ASII (-) variant was also now apparent (middle panel). GAPDH served as a loading control. *N* = 5 for each variant. **(D)** Quantification of RT-PCR data show significant changes in the ASII+/ASII - ratio in *Smn*^*-/-*^;*SMN2* samples compared with controls. The relative levels of adult and neonatal RYR1 isoforms was significantly altered for both the ASI and ASII variants in *Smn*^*2B/-*^ animals compared with controls. **(E**,**F)** The relative levels of adult and neonatal ASII RyR1 transcript variants are not altered in P14 mice one **(E)** and seven **(F)** days post-denervation compared with sham operated mice. *N* = 3.

The results of our physiology experiments led us to investigate possible causes for the decrease in force production from *Smn*^*-/-*^;*SMN2* muscle. During a muscle contraction, calcium is released from the sarcoplasmic reticulum to the sarcomere to allow for the actin-myosin cross-bridge cycling. The calcium release is mediated by ryanodine receptor 1 (RyR1), which is the predominant ryanodine receptor expressed in mature muscle [26]. Several splice variants of the *RyR1* gene exist. For example, one variant is called ASI and is expressed without exon 70 [ASI (-)] in neonatal muscle and transitions to an alternatively spliced variant that includes exon 70 in mature skeletal muscle [ASI (+)] [27]. The second *RyR1* splice variant is ASII, which is further spliced to exclude exon 83 in immature muscle [ASII (-)] or to include that exon in mature muscle [ASII (+)] [21,27]. Using PCR primers designed to target the mature and immature variants, we assessed the RyR1 transcripts in hindlimb skeletal muscle RNA extracts from mouse models of SMA and in controls. A time course analysis demonstrates the predominant expression of ASII (+) in mature muscle (P21) over the ASII (-) variant in wild type mice (Figure 4A). At P5, the predominant ASII isoform in control mice was ASII (+), while in *Smn*^*-/-*^;*SMN2* muscle there was a relative increase in the proportion of the neonatal variant ASII (-) over ASII (+) (Figure 4B middle panel and Figure 4D). In control P21 mice, the adult ASII (+) variant was predominant relative to the neonatal ASII (-) variant, whereas in *Smn*^*2B/-*^ mice both variants were expressed at similar levels (Figure 4C middle panel and Figure 4D).

Using the same approach, we assessed the transcript levels of ASI using primers targeting both the neonatal and adult ASI splice variant. We did not observe any difference in neonatal versus adult transcript levels of the ASI variant for the *Smn*^*-/-*^;*SMN2* mice (Figure 4B upper panel and Figure 4D). In P21 *Smn*^*2B/-*^ mice, however, the neonatal ASI (-) transcript was the predominant variant expressed, while in control muscles, the ASI (+) transcript was the major ASI variant expressed (Figure 4C upper panel and Figure 4D). Collectively, the aberrant expression pattern of the RyR1 transcripts suggests a delay in muscle development in mouse models of SMA.

Muscle denervation at the NMJ is a pathological feature observed in mouse models of SMA. Although there are generally low levels of denervation in the hindlimb muscles of SMA model mice [28], we investigated whether denervation would influence the expression the RyR1 transcript. To do so, we experimentally denervated muscles of 2-week-old wild type mice and examined the expression of RyR1 in denervated samples compared with sham controls. We did not detect any changes in the splicing pattern of the RyR1 ASII variants in denervation compared with control samples, either one day or seven days post-denervation (Figure 4E,F). These results support the hypothesis that the changes in *RyR1* splicing pattern in muscles from the mouse models of SMA are not attributable to pre-synaptic pathology and are therefore potentially reflective of a muscle developmental defect.

### Altered sodium channel levels in SMA mice

In excitable cell types, such as neurons and myocytes, sodium channels propagate the action potential. Sodium channel expression is a developmentally regulated process in which an isoform switch, Na_v_1.5 to Na_v_1.4, occurs during postnatal development in the mouse [29,30]. Na_v_1.4, the predominant sodium channel isoform in adult skeletal muscle [31], must be expressed at the correct time point during development to fulfill its role. A delay in expression of the Na_v_1.4 isoform can negatively impact muscle force production [32].

**Figure 5:**
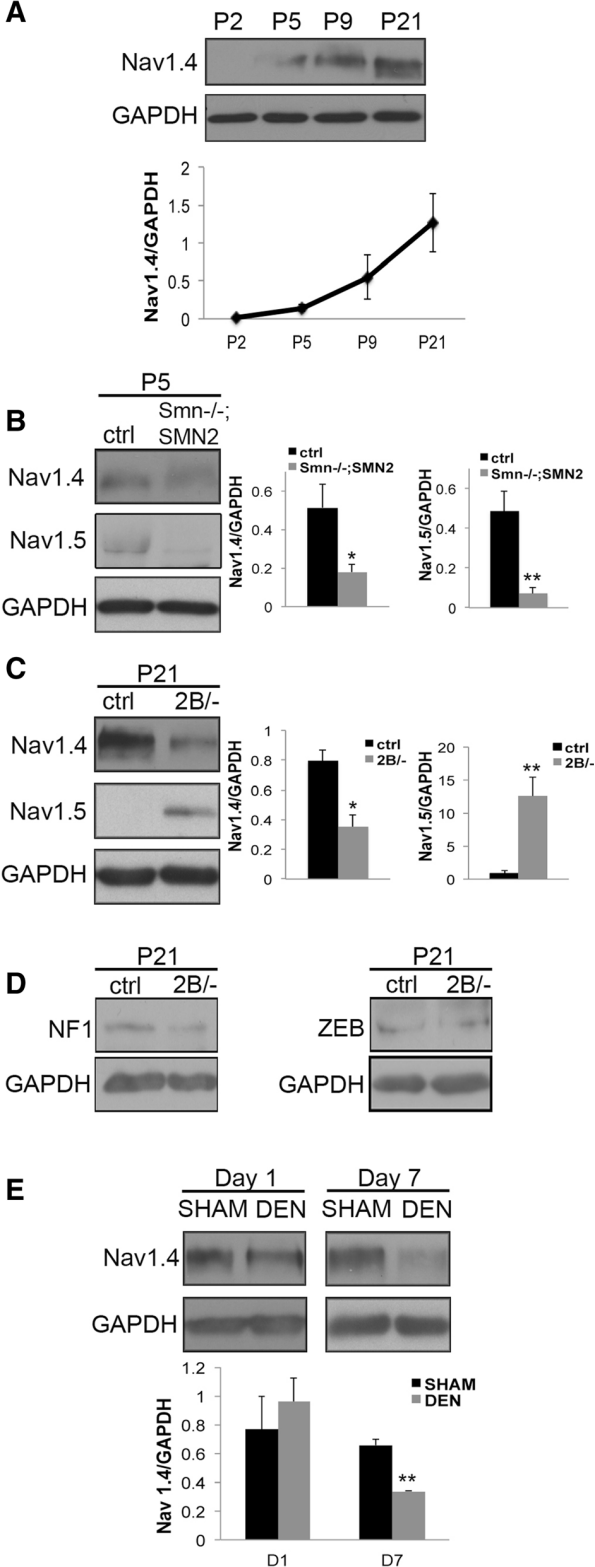
**Na**_**v**_**1.4 protein levels are decreased in muscles from mouse models of SMA. (A)** Immunoblot analysis using muscle lysate from P2, P5, P9, and P21 wild type mice. Na_v_1.4 protein levels increase during postnatal muscle development and form the predominant sodium channel expressed in mature skeletal muscle. GAPDH served as a loading control (*N* = 3). **(B)** Representative immunoblot with quantification, showing a decrease in levels of sodium channel Na_v_1.4 and Na_v_1.5 in P5 *Smn*^*-/-*^;*SMN2* hindlimb muscle compared with controls (*N* = 3). **(C)** Quantification of immunoblot analyses in P21 *Smn*^*2B/-*^ and control hindlimb muscles revealed a decrease in Na_v_1.4 levels. Early in postnatal muscle development, the Na_v_1.5 sodium channel isoform is the most predominant. In P21 *Smn*^*2B/-*^ mice, the protein levels of Na_v_1.5 are higher than in controls (*N* = 3). **(D)** The protein level of the Na_v_1.4 positive regulator, NF1, is not altered in muscles from P21 *Smn*^*2B/-*^ mice. Similarly, no change was detected in the protein levels of the Na_v_1.4 repressor ZEB. **(E)** Expression of sodium channel Na_v_1.4 in control sham and denervated samples 1 and 7 days post-denervation was assessed by immunoblot (*N* = 3). A decrease in the levels of Na_v_1.4 in muscle was noted at 7 days post-denervation. *, *P* < 0.05; **, *P* < 0.01.

As expected, we observed a robust increase in Na_v_1.4 levels in wild type muscle during postnatal development from P2 to P21 (Figure 5A). Interestingly, in two independent mouse models of SMA, there is a decrease in the levels of Na_v_1.4 compared with control mice. Specifically, in P5 *Smn*^*-/-*^;*SMN2* mice, Na_v_1.4 and Na_v_1.5 levels were significantly decreased in hindlimb skeletal muscle compared with control counterparts (Figure 5B). Similarly, in muscle from phenotype stage P21 *Smn*^*2B/-*^ mice, there was a decrease in Na_v_1.4 levels compared with controls (Figure 5C). In addition to the decrease in Na_v_1.4, we observed an increase in Na_v_1.5 levels in *Smn*^*2B/-*^ muscle (Figure 5C). Sodium channel Na_v_1.5 is the predominant isoform expressed in the adult heart and in early stages of skeletal muscle development [30]. These results suggest that muscle development is delayed in SMA model mice and that development is severely impaired, especially in *Smn*^*-/-*^;*SMN2* mice, where both Na_v_ isoform levels are decreased.

To gain a better understanding of how Na_v_1.4 is mis-regulated in SMA mice, we assessed the status of proteins known to regulate sodium channel expression. Hebert and colleagues [32] have previously demonstrated that the transcription factor NF1 is recruited to the *Na*_*v*_*1.4* gene promoter by myogenic regulatory factors to enhance its expression. We did not observe any differences in the levels of NF1 in muscle from P21 *Smn*^*2B/-*^ mice compared with controls (Figure 5D). Another transcription factor, ZEB, is a *Na*_*v*_*1.4* repressor. As with NF1, we did not observe any change in ZEB levels in muscle from *Smn*^*2B/-*^ mice (Figure 5D).

We next investigated whether Na_v_1.4 expression was influenced by experimental denervation. There was no change in Na_v_1.4 levels one day post-denervation (Figure 5E). However, a significant decrease was observed seven days following denervation, in agreement with previous studies [33,34]. Therefore, although the muscles used in the Na_v_1.4 expression analysis are not morphologically denervated, we cannot rule out the possibility that functional synaptic defects at the NMJ influence sodium channel expression in muscles from mouse models of SMA.

### SERCA1a protein expression is altered in *Smn*^-/-^;*SMN2* mice

**Figure 6:**
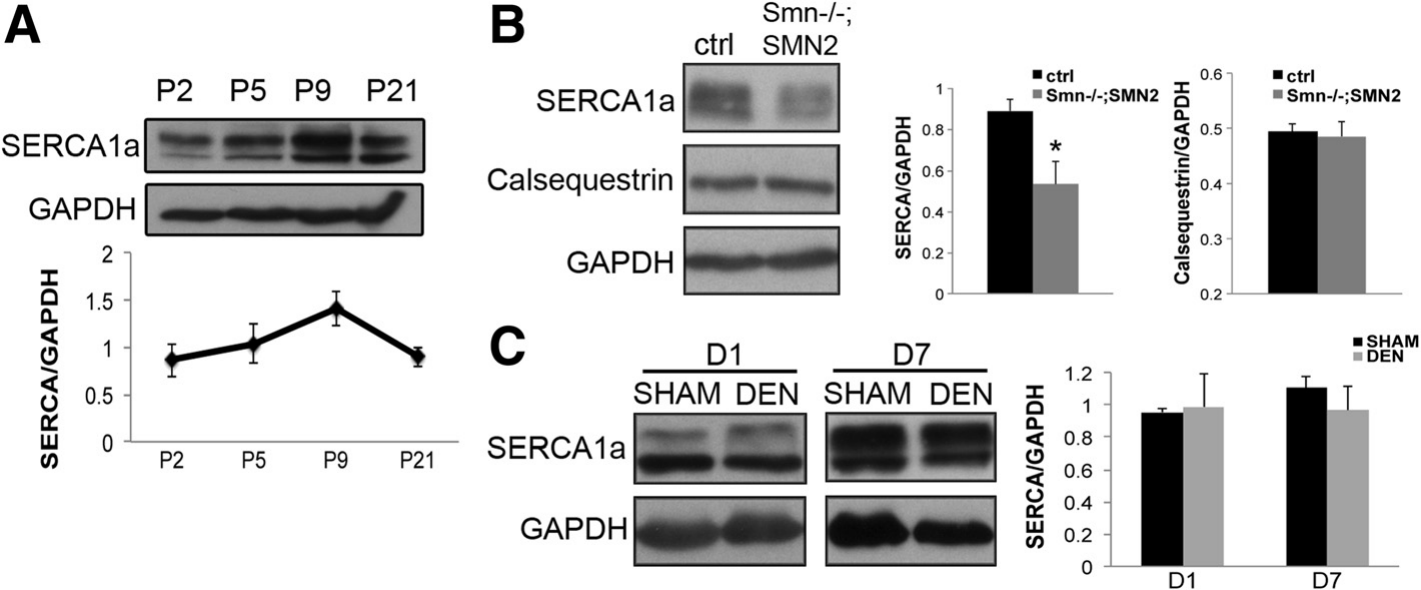
**SERCA1a protein level is altered in muscles from *Smn***^***-/-***^**;*SMN2 *mice. (A)** Whole muscle lysate was collected from P2, P5, P9, and P21 wild type mice and immunoblot analysis was performed to assess SERCA1a protein levels. SERCA1a levels increase over time and peak at P9 (*N* = 3). **(B)** Immunoblot with quantification showing a decrease in SERCA1a, but not calsequestrin, in hindlimb muscle from P5 *Smn*^*-/-*^;*SMN2* mice compared with control (*N* = 3). **(C)** Immunoblots were performed on muscle lysates collected from experimentally denervated (DEN) and sham operated (SHAM) muscle. No change in SERCA1a levels was observed. *N* = 3, *, *P* < 0.05.

One possible mechanism that can cause increased unstimulated force production is an incomplete removal of Ca^2+^ from the sarcoplasm because of decreased levels of the Ca^2+^ ATPase pump. The protein responsible for the Ca^2+^ uptake following a muscle contraction is the sarcoplasmic reticulum Ca^2+^ ATPase (SERCA), of which SERCA1a is the predominant isoform found in fast-twitch muscles, such as the TA muscle [35]. The protein expression of SERCA1a is developmentally regulated. It peaks by P9 and drops slightly at P21 (Figure 6A). Immunoblot analysis revealed a decrease in SERCA1a protein levels in hindlimb skeletal muscles from P5 *Smn*^*-/-*^;*SMN2* mice compared with control samples (Figure 6B). Interestingly, levels of calsequestrin, a protein that binds and stores Ca^2+^ in the sarcoplasmic reticulum, was unchanged in *Smn*^*-/-*^;*SMN2* muscle compared with controls (Figure 6B), indicating that a Ca^2+^ handling defect was likely limited to the sarcoplasmic reticulum pump.

Next, we measured the influence of denervation on SERCA1a protein levels. Protein lysate from gastrocnemius muscles was collected from denervated and sham operated mice. SERCA1a protein levels were unchanged in skeletal muscle from denervated mice compared with controls (Figure 6C). This again supports the hypothesis that the observed decrease in SERCA1a in muscle from *Smn*^*-/-*^;*SMN2* mice could be due to a muscle developmental defect.

## Discussion

Here, we show that in two mouse models of SMA, muscle weakness occurs early, being evident prior to any overt physical denervation and motor neuron loss. This physiological defect was associated with delayed expression of mature isoforms of proteins important for muscle function. Our results therefore point to muscle weakness coupled with delayed muscle development and provide new insight into the pathophysiology underlying SMA. This work highlights the potential of muscle as a therapeutic target and warrants further work to identify muscle directed strategies to increase muscle force production.

### Muscle weakness in SMA mice

We have employed an *ex vivo* method in which the muscle is excised and placed in a chamber where it can be directly stimulated to contract. By doing so, we reduce the negative contribution that degenerating motor neurons might have in eliciting a contraction, with the caveat that there may still be functional defects preceding the analysis. We show a decrease in normalized peak tetanic force in muscle from phenotype stage *Smn*^*-/-*^;*SMN2* mice. Importantly, we show a similar decrease in muscle force from pre-symptomatic *Smn*^*-/-*^;*SMN2* and *Smn*^*2B/-*^ mice prior to any overt motor neuron loss and denervation, although we cannot rule out the influence of a functional deficit within the motor neurons. It should be noted, however, that our physiological results were normalized to the cross-sectional area of each muscle tested. Therefore, the overt decrease in muscle size observed in P5 *Smn*^*-/-*^;*SMN2* mice cannot explain the decrease in force production, *per se*. In addition, our experiments performed on pre-symptomatic mice allow us to rule out the possibility that smaller myofibers are the reason for the decrease in relative force production, since no significant difference was observed in muscle size between pre-symptomatic and control mice. However, the maturity of the muscle may influence force production, irrespective of size. As we have observed a decrease in the mature isoforms of a number of muscle proteins, we suggest that a decrease in muscle maturity in P2 *Smn*^*-/-*^;*SMN2* and P9 *Smn*^*2B/-*^ mice could contribute to a marked decrease in force production.

### Delayed expression of mature isoforms of muscle function proteins in mouse models of SMA

Several groups have indirectly demonstrated impaired muscle growth in mouse models of SMA by measuring the cross-sectional area of developing myofibers [18-20]. These analyses suggest that shortly after birth, muscle development is significantly impaired. During postnatal muscle development, as myotubes grow to become myofibers, a switch in expression from neonatal to adult protein isoforms occurs for many muscle function proteins. A delay in this switch could compromise muscle maturation and function. Such might be the case with the expression of MHC, in which the embryonic and perinatal MHC isoforms are predominantly expressed in muscle from SMA model mice [19,20]. Therefore, we hypothesized that several other proteins important for generating muscle contractions could be aberrantly expressed, with juvenile isoforms predominating rather than adult ones, which could lead to muscle weakness in mouse models of SMA. We focused on proteins that are directly involved in the regulation of muscle contraction, that is, proteins important for calcium regulation and action potential propagation.

### RyR1 expression in muscle from mouse models of SMA

Results from our RT-PCR analysis revealed a delay in the expression of the mature *RyR1* splice variants in skeletal muscle from mouse models of SMA. In phenotype stage *Smn*^*2B/-*^ mice, we observed a mis-regulation of both the ASI and ASII alternatively spliced variants. At P5 in the *Smn*^*-/-*^;*SMN2* model, a change in expression was evident for the ASII variant but not the ASI. During development, the transition from ASII (-) to ASII (+) begins at P0 and is complete by P21 [27]. For the ASI variant, the transition from the neonatal ASI (-) to the adult ASI (+) form begins only at P8. Therefore, the timing of the ASI transition probably explains why we observed the delay in P21 *Smn*^*2B/-*^ mice but not in P5 *Smn*^*-/-*^;*SMN2* mice. The functional studies performed by Kimura *et al.* demonstrate that neonatal RyR1 is less active than adult RyR1, as it binds ryanodine with less affinity than the adult form, and therefore releases less calcium [21]. Thus, the persistent expression of the neonatal RyR1 variants in mouse models of SMA probably leads to decreased Ca^2+^ release from the sarcoplasmic reticulum to the sarcomere, and subsequently results in weaker muscle contractions.

### Sodium channel expression in muscle from mouse models of SMA

In skeletal muscle, action potentials are generated and propagated by voltage-gated sodium channels. Na_v_1.4 is the predominant pore-conducting channel in adult muscle. Its expression significantly increases in mice in the first two weeks after birth [29,31]. Here we show that Na_v_1.4 levels are decreased in muscles from two different mouse models of SMA. This may explain in part the lower force generation, since there would have been an insufficient number of available Na_v_1.4 channels to generate action potentials during a train. Furthermore, this period after birth coincides with a period of dramatic muscle growth, and Na_v_1.5 is the major sodium channel expressed during early muscle development. Upon denervation of skeletal muscle, the expression of sodium channels reverts to that which occurs during development [31]. The expression of Na_v_1.5 increases and that of Na_v_1.4 decreases in denervated muscle. Indeed, we observed a decrease in Na_v_1.4 levels in experimentally denervated muscles (day 7), as well as in muscles from both SMA mouse models studied. As such, we cannot rule out the possibility that the mis-regulation of Na_v_1.4 is due to denervation in muscle from the symptomatic mice.

The expression of *Na*_*v*_*1.4* is positively regulated by the transcription factor NF1 and is repressed by the transcription factor ZEB [32]. We did not observe any differences in the expression of these two transcription factors in *Smn*^*2B/-*^ mice. The recruitment of the NF1 protein to the *Na*_*v*_*1.4* promoter is mediated through two transcription factors that are important for muscle differentiation, namely myogenin and muscle-specific regulatory factor 4 (MRF4). It can be envisaged that a delay in the expression of myogenic regulatory factors, such as myogenin and MRF4, or others even more up-stream of myogenin and MRF4, may explain the deferred Na_v_1.4 expression in SMA mice.

### Decreased SERCA1a expression in *Smn*^-/-^;*SMN2* mice

The results from our fatigue protocol demonstrate an increase in unstimulated force and a decrease in the time of unstimulated force onset in *Smn*^*-/-*^;*SMN2* mice. This observation may be indicative of a defect in Ca^2+^ uptake from the sarcomere to the sarcoplasmic reticulum, which is supported by the muscle intrinsic decrease in levels of the SERCA1a Ca^2+^ pump in muscles of *Smn*^*-/-*^;*SMN2* mice. Defects in Ca^2+^ handling have previously been reported in mouse models of muscular dystrophies [21,36]. Specifically, defects related to Ca^2+^ uptake and SERCA1 function have been described in a mouse model of Duchenne’s muscular dystrophy [37]. Indeed, the overexpression of SERCA1 in skeletal muscles led to robust improvements in muscle function and attenuated muscle pathology in mouse models of muscular dystrophy [38]. Furthermore, *RyR1* splicing defects resulting in the expression of the neonatal variants contribute to the pathogenesis of the neuromuscular disease myotonic dystrophy type 1 [21]. Thus, the defects we report in muscles from SMA model mice are reminiscent of those that occur in other muscle diseases.

## Conclusions

In summary, we have demonstrated early and profound muscle weakness, and aberrant expression of muscle proteins in two different mouse models of SMA, which may contribute to the SMA phenotype. Our results provide significant insight into muscle defects in SMA pathophysiology and suggest that including skeletal muscle as a therapeutic target in SMA is warranted.

## Abbreviations

GAPDH: Glyceraldehyde-3-phosphate dehydrogenase; H & E: Hematoxylin and eosin; MHC: Myosin heavy chain; MRF4: Muscle-specific regulatory factor 4; NF: Neurofilament; NF1: Nuclear factor 1; NMJ: Neuromuscular junction; P: Postnatal day; PCR: Polymerase chain reaction; RT-PCR: Reverse-transcription polymerase chain reaction; RyR1: Ryanodine receptor 1; SERCA: Sarcoplasmic reticulum Ca2+ ATPase; SMA: Spinal muscular atrophy; SMN: Survival motor neuron; SV2: Synaptic vesicle protein 2; TA: Tibialis anterior; ZEB: Zinc-finger E box-binding protein.

## Competing interests

The authors declared that they have no competing interests.

## Authors’ contributions

JGB and RK conceived and designed the project. JGB performed and analyzed most of the experiments and was assisted by KS for Figures 1 and 3, and by YDR for panels A and C of Figure 2. LMM performed experiments and analyzed the data in panels E-H of Figure 2. JMR supervised KS and helped analyze the data in Figure 1. JGB wrote the paper and RK revised and edited it. All authors read and approved the final manuscript.
